# Codesign and knowledge translation of the Strength-based, Tiered, Accessible Resources and Supports (STARS) for Kids study to identify and support child development, parental mentalwell-being and family psychosocial needs: a mixed-methods research protocol

**DOI:** 10.1136/bmjpo-2025-004031

**Published:** 2026-05-07

**Authors:** James R John, Hayley Robinson, Sini Lambiase, Sophie Nicholls, Maite Diez, Karlen R Barr, Katarina Ostojic, Aunty Kerrie Doyle, Luana Kliendienst, Melissa Foster, Lynette Syron, Toni Carson, Jane Kohlhoff, Ann Dadich, Clare Brennan, Bree Katsamangos, Lynn Kemp, Amy Finlay-Jones, Grainne O’Loughlin, Katherine Boydell, Shanti Raman, Nicole Deen, Kenny Lawson, Virginia Schmied, Ilan Katz, Andrew Page, Rebekah Grace, Anna-Marie Kanaan, Rebecca Young, Erin Brandtman, Lee Bratel, Michael Hodgins, Raghu Lingam, Christa Lam-Cassettari, Sharon Goldfeld, Adam K Walker, Ping-I Lin, Susan Morton, Desiree Silva, Jenny Downs, Susan Woolfenden, Valsamma Eapen, Ngarra Wakulda Aboriginal Community Consultation Group, Aunty Isabell Bungie, STARS for Kids author group, Paul Saunders

**Affiliations:** 1 School of Clinical Medicine, University of New South Wales, Sydney, New South Wales, Australia; 2 Ingham Institute for Applied Medical Research, Liverpool, New South Wales, Australia; 3 Academic Unit of Infant, Child and Adolescent Psychiatry Services, South Western Sydney Local Health District, Liverpool, New South Wales, Australia; 4 The Kids Research Institute Australia, Nedlands, Western Australia, Australia; 5 Faculty of Medicine and Health, The University of Sydney, Sydney, New South Wales, Australia; 6 Indigenous Health, Western Sydney University, Sydney, New South Wales, Australia; 7 School of Rural Health, Charles Sturt University, Orange, New South Wales, Australia; 8 Biripi Aboriginal Corporation Medical Centre, Purfleet, New South Wales, Australia; 9 First Steps Count, Taree, New South Wales, Australia; 10 Karitane, Villawood, New South Wales, Australia; 11 School of Business, Western Sydney University, Penrith, New South Wales, Australia; 12 Mission Australia, Taree, New South Wales, Australia; 13 School of Nursing and Midwifery, Western Sydney University, Sydney, New South Wales, Australia; 14 Faculty of Health Sciences, Curtin University, Perth, Western Australia, Australia; 15 Black Dog Institute, Sydney, New South Wales, Australia; 16 Community Paediatrics, South Western Sydney Local Health District, Liverpool, New South Wales, Australia; 17 Australian Research Alliance for Children and Youth, Canberra, New South Wales, Australia; 18 Translational Health Research Institute, Western Sydney University, Penrith, New South Wales, Australia; 19 Social Policy Research Centre, Faculty of Arts, Design and Architecture, University of New South Wales, Sydney, New South Wales, Australia; 20 TeEACH Strategic Research Institute, Western Sydney University, Penrith, New South Wales, Australia; 21 Murdoch Childrens Research Institute, Melbourne, Victoria, Australia; 22 Department of Paediatrics, The University of Melbourne, Melbourne, Victoria, Australia; 23 Neuroscience Research Australia, Sydney, New South Wales, Australia; 24 Research Institute for Innovative Solutions for Wellbeing and Health, University of Technology Sydney Faculty of Health, Sydney, New South Wales, Australia; 25 The Kids Research Institute Australia, Perth, New South Wales, Australia; 26 Joondalup Health Campus, Perth, Western Australia, Australia; 27 Curtin School of Allied Health, Curtin University, Perth, Western Australia, Australia; 28 Institute for Women, Children and their Families, Sydney, New South Wales, Australia

**Keywords:** Child Health, Health services research, Mothers, Qualitative research

## Abstract

**Introduction:**

Many children and their families, especially those from priority populations, experience barriers to accessing high-quality early childhood health, education, social and legal services. Further, these families are often under-represented in service planning and research; hence innovations are not designed to meet their needs. Our aim is to codesign with families and the wider community, a Strength-based, Tiered, Accessible Resources and Supports for Kids (STARS for Kids) programme to optimise child development, parental mental well-being, and family psychosocial needs in the first 2000 days from pregnancy to start of school.

**Methods and analysis:**

This study will employ a mixed methods design at three sites: (1) Fairfield, urban multicultural site in South-Western Sydney New South Wales; (2) Taree, a regional town with a large Indigenous community; and (3) The City of Wanneroo, a low socioeconomic area of Western Australia. The codesign process will involve five phases of the design thinking methodology informed by culturally safe, strengths-based, and trauma-informed practices. Codesign will involve families, service providers, and community leaders from priority groups such as multicultural stakeholders from South-Western Sydney and an Aboriginal Community Consultation Group with Biripi Elders and other local Indigenous representatives at Taree. Data collection will include semi-structured interviews, workshops, or focus groups as well as ‘yarning’ for the Aboriginal community. Qualitative data will be thematically analysed using Braun and Clarke’s six-phase method of thematic approach.

**Trial registration number:**

Australian New Zealand Clinical Trials Registry - ACTRN12624000806561 (This protocol pertains only to the initial codesign phase, during which the STARS for Kids tiered care model will be finalised for subsequent implementation and evaluation in the next trial phase which is outlined in the trial registry).

**WHAT IS ALREADY KNOWN ON THIS TOPIC**

**WHAT THIS STUDY ADDS**

- The ‘STARS for Kids’ study will codesign a tiered model of care to identify and provide ‘whole of family’ support for child development, parental mental health, and psychosocial needs with priority population groups in the first 2000 days across urban and regional sites in Australia. A key strength is the active involvement of families, service providers, and community leaders to ensure diverse voices inform programme development.

**HOW THIS STUDY MIGHT AFFECT RESEARCH, PRACTICE OR POLICY**

- The ongoing iterative codesign process across the study period will ensure the data is used to inform the local community response with recommendations for action. Additionally, the local and overarching knowledge translation component will ensure that findings from the ‘STARS for Kids’ study contribute to ongoing quality improvement efforts. By synthesising key information, the study can generate evidence to support decision-making to optimise policy and practice in the first 2000 days across communities in Australia.

## Background

### The need to support equity in early childhood

 The first 2000 days of a child’s life, from conception to start of school, is a critical time to ensure all children have the best possible start in life.^1 2^ However, children from priority populations (including Indigenous, culturally and linguistically diverse (CALD), rural/regional and/or those who experience socioeconomic adversity) often experience financial, structural, and systemic barriers in accessing and benefitting from early childhood health, education, and social supports.^3 4^ This is further compounded by the Australian child and family service system’s complexity and inefficiencies, including fragmentation, duplication, and service gaps^5 6^ resulting in missed opportunities for early identification and intervention.^5 6^ Although the benefits of early interventions are well evidenced,^7^,^9^ systematic mechanisms to engage children and families early are still lacking, as reflected in the low and inequitable completion of developmental checks beyond 6 months of age.^10^,^13^ In addition, poor parental mental health can also result in adverse child developmental trajectories^14^ leading to intergenerational cycles of adversity and reinforcing broader societal inequities.^15^

While several national and state policies have highlighted the need to promote integrated services to address inequitable developmental and health outcomes in the mainstream service system, healthcare planning and related research, the voices of families from priority populations are often under-represented.^16 17^ Consequently, service innovations might not meet their needs.^18^ Further, Aboriginal and Torres Strait Islander families and CALD communities have had fewer opportunities to contribute to the design and development of research models that effectively use their strengths and ensure cultural safety.^19 20^ A fundamental shift is required from reactive, siloed services towards a proactive and holistic approach, codesigned and led by priority populations. Additionally, research on the first 2000 days of life has largely focused on risks such as adverse childhood experiences,^21^ but emerging evidence highlights their limitations for identifying individual vulnerabilities,^22^ prompting a shift towards strengths-based approaches to better support families and improve long-term outcomes. Strengths-based programmes recognise and build on the existing capacities, resilience, cultural knowledge, and resources of children, families and communities, thereby emphasising empowerment, partnership and respect for lived experience in the design and delivery of supports.^23 24^

### Current evidence on integrated child and family models

Integrated digital and place-based models of care can promote child development, parental mental well-being, and family psychosocial needs. Further, digital solutions to enhance early identification via efficient developmental surveillance^25 26^ and in-person, place-based hub models of care for integrated service delivery can facilitate improved outcomes and cost-benefits.^27^,^29^ While there is some international evidence suggesting the benefits of place-based programmes such as the UK’s Sure Start programme,^29^ none have used an integrated hybrid (digital and/or place-based) programme with a ‘whole of family’ approach, which are codesigned with the families.

### Working with and building on the strengths of families, communities and services

Codesign is a collaborative process wherein a diverse group of participants actively engage in exploring, creating, and testing solutions to common challenges.^30^ Effective codesign relies on knowledge translation, ensuring that research evidence, lived experiences, and community insights are integrated into practical, culturally responsive solutions^31^ and promote a more equitable service access and supports, regardless of their cultural, geographical and socioeconomic backgrounds.^32^

### Aims and objectives

This study aims to codesign a Strength-based, Tiered, Accessible Resources and Supports (STARS) programme for Kids^33^ with the goal to systematically identify early and facilitate tailored supports for child development, parental mental health, and family psychosocial needs. This will be achieved by: (1) understanding parents’, carers’, community members’ and service providers’ experiences of, priorities for, and challenges in the child and family service journey; (2) codesigning solutions for early identification and intervention via hybrid (digital and/or place-based) integrated tiered care model; and (3) identifying the key components of a hybrid responsive, integrated, sustainable, and equitable tiered care model through a comprehensive and ongoing knowledge translation process.

Once codesigned, our ultimate aim is to implement and evaluate the codesigned STARS for Kids in empowering parental engagement with developmental surveillance as well as provide tailored supports for child development, parental mental health, and family psychosocial needs.

## Methods and analysis

### Study design

**Figure 1:**
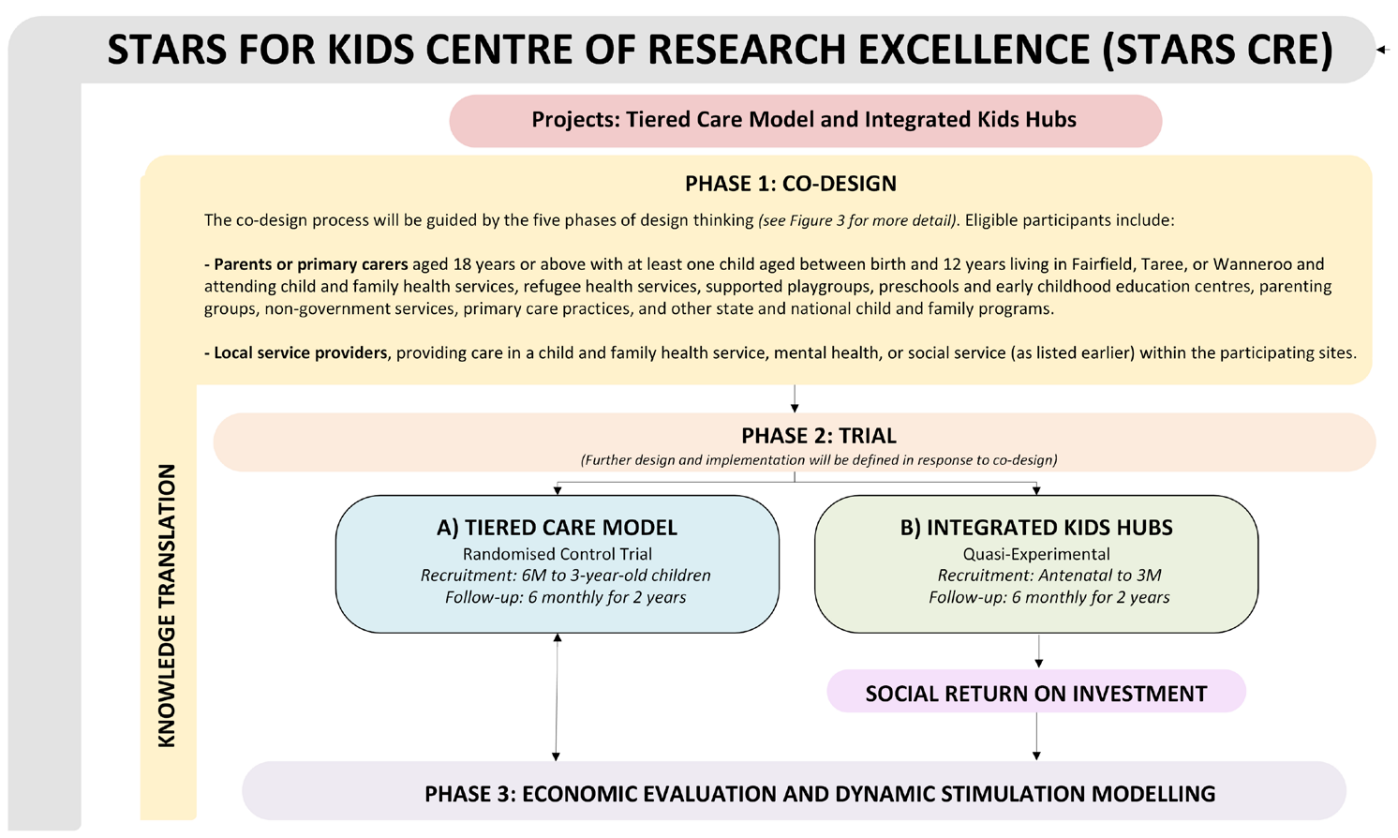
Overview of the STARS for Kids programme. CRE, Centre for Research Excellence; STARS, Strength-based, Tiered, Accessible Resources and Supports.

We will employ a mixed methods codesign approach to develop the STARS for Kids model prototype, which will be evaluated in a multisite randomised controlled trial (see figure 1).

### Study setting

This is a multisite study conducted in three sites in Australia: Fairfield, an urban community in South Western Sydney (SWS), New South Wales (NSW) (56% of Fairfield residents are born overseas compared with the NSW state average of 34%); a regional/rural community in Taree in NSW with a large Indigenous population (about 5.6% of the population compared with NSW state average of 3.4%); and low socioeconomic status suburbs in the City of Wanneroo with Index of Relative Socio-economic Advantage and Disadvantage score below the national average of 1000 (eg, Koondoola, Girrawheen) in Western Australia. These sites were chosen as they represent significant disadvantage, and identified as most impacted areas via a national survey of the top 10 communities where children experienced family employment stress during the COVID-19 pandemic.^34^ Additionally, in the hub sites - Taree and Fairfield, one in four children are developmentally vulnerable at the start of school as per the Australian Early Development Census, with rates in some areas within our sites as high as one in three against national average of one in five.^35^

### Study participants and advisory groups

Eligible participants include:

- Parents or primary carers aged 18 years or above with at least one child aged between birth and 12 years living in Fairfield, Taree, or Wanneroo and attending child and family health services, refugee health services, supported playgroups, preschools and early childhood education centres, parenting groups, non-government services, primary care practices, and other state and national child and family programmes.
- Local service providers, providing care in a child and family health service, mental health, or social service (as listed earlier) within the participating sites.

The codesign process will be facilitated by an expert in co-design with a co-facilitator from the oversight advisory group of stakeholders including parents/carers and service providers at each site, and Ngarra Wakulda Aboriginal Community Consultation Group (ACCG) at Taree to guide the codesign and research processes, and ensure cultural safety and data sovereignty for the community.

### Recruitment and consenting process

#### Parent/carer participants

**Figure 2:**
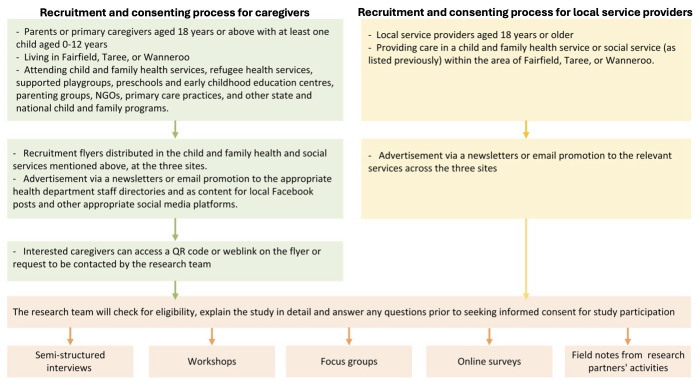
Flowchart of recruitment process. NGOs, non-governmental organisations; QR, quick response.

The study will be promoted using flyers/posters in the reception areas of the relevant services at the three sites (see figure 2) as well as relevant newsletters, email, or social media platforms in the community. To minimise under-representation of families who are traditionally described as ‘hard to reach’, recruitment will be supported by attendance at community outreach programmes or in-person engagement within trusted community hub providers and organisations, Aboriginal Community Controlled Organisations, and frontline service providers. Additionally, flexible participation options (in-person, telephone, and online), alongside the use of interpreters where required, will be employed to support inclusive participation.

Given the substantial proportion of multicultural communities in SWS, flyers/posters will be translated in Chinese, Arabic, and Vietnamese, alongside interpreter services if needed. Additionally, the flyers/posters will be codesigned with the Ngarra Wakulda ACCG in Taree and advertised via relevant services and organisations.

Parents/carers attending the aforesaid services at the sites who are interested in the study can access a QR (quick response) code or weblink on the flyer/poster or be linked up with the research team by service staff. After the researchers have contacted and ensured the eligibility, a participant information sheet and consent form (PISCF) with detailed study information, will be provided via email (QR code/weblink), in-person (paper-based form), or telephone (verbal explanation), depending on the prospective participant’s preference.

#### Local service provider participants

The research team will recruit local service providers from the relevant services across the three sites via self-nomination or by researchers’ information shared via email.

### Consenting process

Participants will be given time to decide whether and how they would like to participate in the study and they will be able to provide informed consent in written form, electronically, or verbally. Consent will be voluntary and free from coercion.

### STARS for Kids conceptual model

The STARS for Kids conceptual model uses a tiered model of care that includes digital and place-based Hubs. This comprises the universal tier involving the Watch Me Grow-Electronic (WMG-E)^26 36^ platform, a digital data collection tool, that will be used by parents/carers to complete a series of assessments relating to their child’s developmental needs, parental/carer mental well-being, and family psychosocial needs. The WMG-E will reach families in their homes or in the community on the principle of ‘going to where the children and families go’ and leveraging opportunistic service contacts (eg, childhood immunisation that has 95% reach/uptake), facilitated through trusted providers (eg, primary care, multicultural, refugee or Aboriginal services and other community services such as libraries and playgroups).^37^

Based on their responses, the WMG-E platform will triage them to the appropriate additional or targeted tiers of support and ongoing monitoring. Throughout their engagement with the WMG-E platform, automated reminders will be used to alert families when the next relevant developmental checks are due. The tiers of support will include: Tier 1 (eg, light touch services, signposting and anticipatory guidance); Tier 2 (eg, targeted support for mild to moderate risk) and Tier 3 (eg, wraparound support for those with complex psychosocial needs by service navigators operating in digital or place-based Hubs). The WMG-E has demonstrated feasibility for engaging and empowering priority populations,^26 38^ acceptability for service providers^39^ and effectiveness in identifying developmental conditions including autism^40^ with high sensitivity (94% against gold-standard assessment). The codesign process will inform the adaptation of the platform and the tiers, according to the needs of each site and community, the services available and the health service context.

### Steps in codesigning the STARS for Kids programme

**Figure 3:**
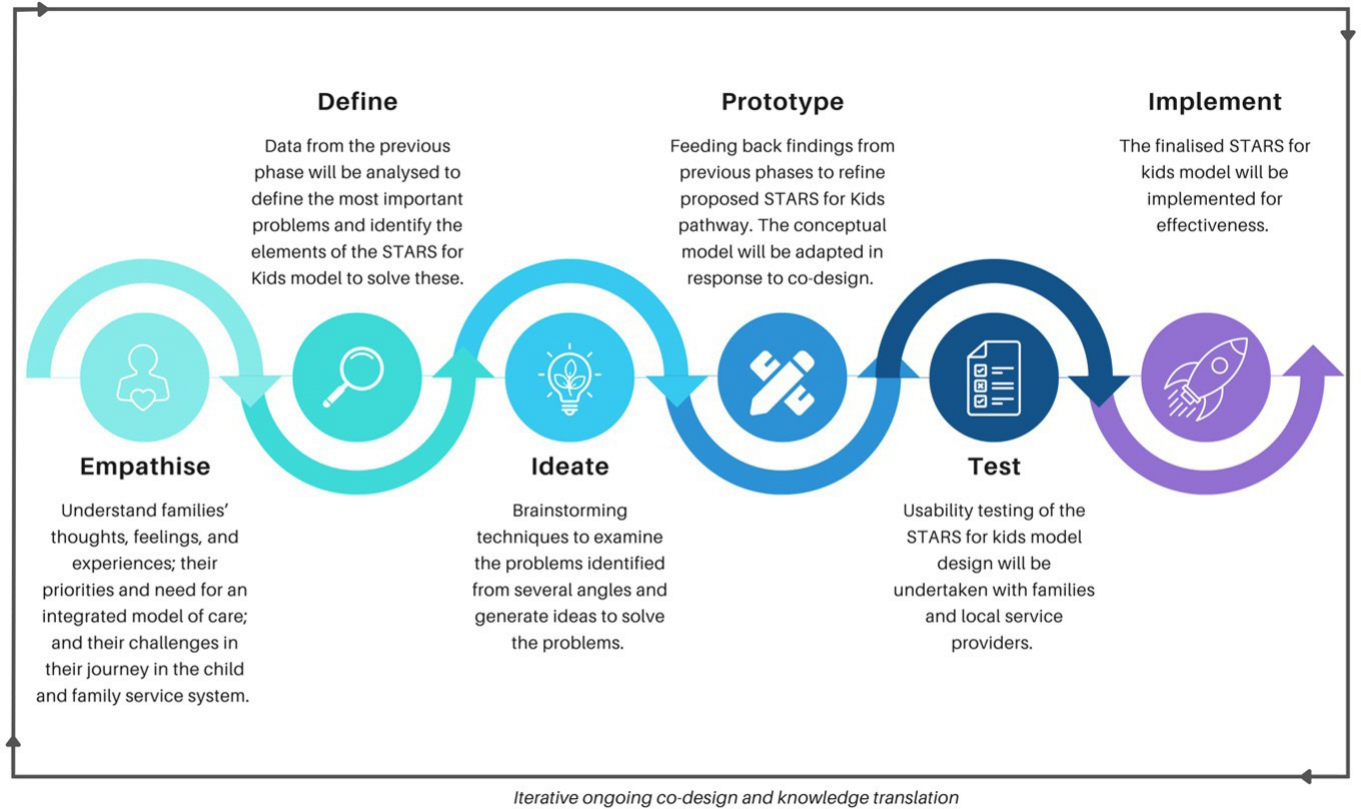
Steps in the iterative codesign process. STARS, Strength-based, Tiered, Accessible Resources and Supports.

The codesign process will be undertaken using the five phases of design thinking, a ‘human-centric’ approach that involves collaboratively developing best-fit solutions with families and key stakeholders (see figure 3).^41^ Specifically, it involves: empathising; developing a clear and succinct problem definition; creative ideation; prototyping; and testing a new solution that can be deployed quickly and cost-effectively.^41^

#### Phase 1: empathise with users

The ‘Empathise’ phase serves to understand parents’/carers’ thoughts, feelings, and experiences. These will be focused on understanding: the child’s developmental needs; challenges in the journey from identification to addressing developmental concerns in the child and family service system; and priorities and need for an integrated model of care. This will be achieved through semi-structured interviews, yarning, and/or focus group with parents/carers, in person, via telephone, or via a secure web-conferencing platform (pending participating preference), complemented by fieldwork notes. Yarning is an Indigenist research method that involves informal, narrative-based conversations with Aboriginal and Torres Strait Islander families that is culturally appropriate and grounded in relationality, trust-building, and active listening.^42^

### Phase 2: defining needs, problems and strengths

The ‘Define’ phase will involve synthesising the insights gathered during phase 1 to formulate a clear and actionable problem statement for the STARS for Kids programme. The findings from phase 2 will be presented as a lay summary with infographics. This output will be shared with participants via workshops and newsletters across the study sites to ensure it defines their needs and problems.

### Phase 3: creating new ideas

The ‘Ideate’ phase will involve generating potential solutions to address the defined challenges within the STARS for Kids framework such as developing a prototype tailored to specific needs, and are culturally safe. This phase will draw on evidence-based practices identified through mapping existing local services and consultation with clinical, community, and policy experts, and integrating these with new insights to develop a tiered model of care.

### Phase 4: prototype new solutions

**Figure 4:**
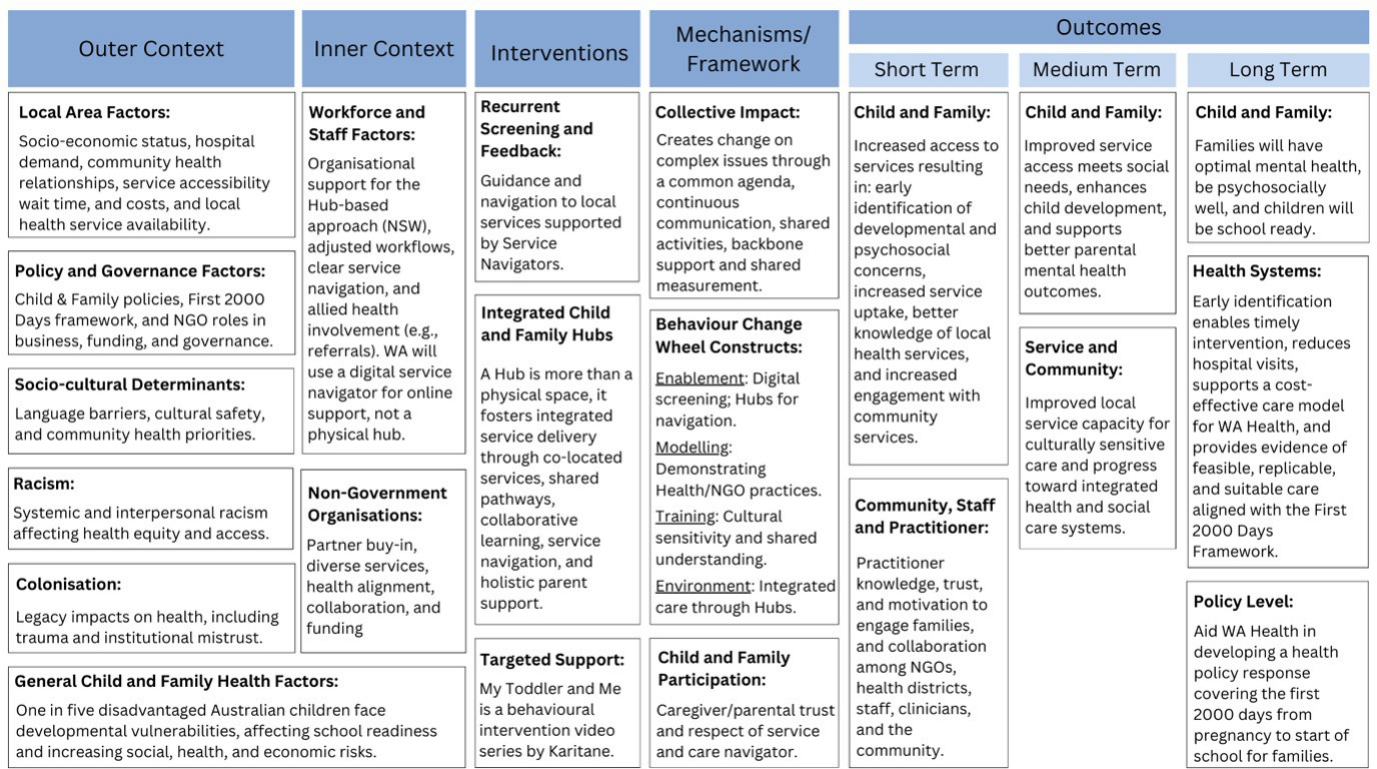
STARS for Kids programme logic model. NGOs, non governmental organisations; NSW, New South Wales; STARS, Strength-based, Tiered, Accessible Resources and Supports.

In the ‘Prototype’ phase, the ideas generated during the ideation phase will be transformed into a final model for the STARS for Kids programme building on the current conceptual model and further refining through iterative testing and refinement (see logic model, figure 4). The logic model based on the Consolidated Framework for Implementation Research (CFIR), outlines the factors influencing programme implementation, its key interventions and the intended outcomes.^43 44^ This model integrates elements from the outer context, such as local area factors and policy considerations, with inner context elements, like workforce factors and non-government organisations’ roles. The logic model also highlights the mechanisms to achieve these outcomes including: collective impact and behaviour change constructs, ensuring that the programme components are responsive to the identified needs and can be scaled appropriately.

### Phase 5: testing

The STARS for Kids draft logic model, clinical pathway, core components, standard operating procedures for the service navigators, and relevant referral resources (detailed site-specific service maps) will be evaluated by the researchers, steering committee, and research advisory groups. Using an iterative Plan-Do-Study-Act (PDSA) approach,^45^ the tiered care components including digital and place-based services will be tested, refined, and integrated as part of a cohesive ecosystem. Insights from these cycles will inform adjustments to the logic model, clinical pathway, and core components. Following this iterative testing and refinement, the final logic model, pathway and tier components, including the Hubs, will be formalised into a manual for the implementation and evaluation phase of the STARS for Kids programme.

### Iterative ongoing codesign and knowledge translation

Throughout the evaluation of the pathways and tier components, the researchers will gather anonymous data on experiences and satisfaction from the participants and will use PDSA iterative cycles. The stakeholders will be actively supported to participate in monthly online/in-person forums, and engage in regular communication using telephone, text and/or email, as per participant choice and convenience. The purpose of these exchanges will be to: identify key service touchpoints and improvements; determine how each component within the system interacts; and review the tiered care pathway including digital/place-based Hub model. Additionally, semi-structured interviews with the Hub service providers and their managers (approximately 5–10 participants per digital/place-based Hub) will be conducted at the beginning, midpoint, and end of the study to ascertain project outcomes and evaluate the acceptability, appropriateness, and feasibility.^46^ At the Taree site, when working together with Indigenous participants, we aim for the co-design to be a healing process of truth-telling and an investment in building trust, hearing stories and responding to ideas and suggestions, as well as acknowledging difficult past experiences.

After the evaluation phase, semi-structured interviews will be carried out to determine the perceived value of the model. To visualise service experiences, the interviewees will be invited to bring an artefact to the interview that may capture their: past experiences; ideal vision that epitomises integrated care via digital/place-based Hubs to them (eg, material objects, photographs, equipment, artwork); or their experience with codesign, reflecting a modified form of photovoice; discuss why they chose the artefact and what it represents to them; and map client journeys to ascertain patterns and clarify how the service system responds (or can respond to) family needs and preferences.^47^ The researchers will then work with artists to develop an online artwork exhibition of participant artefacts, publicly accessible via an online resource. The purpose of this exhibition is to promote knowledge translation, given that arts-based approaches can help to communicate that which is sometimes beyond words.^48^

### Data collection

#### Demographic information

Data collection will include, but not be limited to, demographic information from participating parents/carers, such as age, gender, ethnicity, Aboriginality (in line with site specific Aboriginal Health and Medical Research Council (AH&MRC) Ethics Committee approval), postcode, education, employment status, and the age of their child. Participating service providers will be asked to indicate their role, organisation, and years of experience.

#### Semi-structured telephone/online interviews, workshops, focus groups, yarning and/or online surveys

The interviews will be guided by an open-ended interview schedule, complete with prompting questions. Interviews will be estimated to range between 30 and 60 min to minimise participant inconvenience. Before each interview, participants will be reminded that their contribution will be confidential and that identifying information will be removed from study outputs. At the Taree site, yarns will focus on multiple cycles of narrative-based discussion. This includes creating a setting for truth-telling, active listening, and acknowledgement. The stories gathered from Aboriginal and/or Torres Strait Islander participants will be analysed only by researchers working on that site, to ensure the context is maintained and the inclusion of the Ngarra Wakulda ACCG. Specifically, the interviews will be conducted in person to support relationship building, connection, and cultural safety, supplemented with field observations and satisfaction questionnaires.

### Sample size

Recruitment will continue until thematic saturation is reached. Following previous research, approximately 10–20 participants per group is expected to be sufficient.^49^ Additionally, semi-structured interviews with approximately 5–10 participants from Hub service providers and their managers (per Hub) will be conducted at the beginning, midpoint, and end of the study. These interviews will include: the legal needs assessment, exploration of the Hubs acceptability, appropriateness and feasibility, and achievement of study outcomes.

### Analysis plan

The digital recordings will be transcribed verbatim and thematically analysed using NVivo V.12. Braun and Clarke’s six-phase method of inductive reflexive thematic analysis will be implemented to ensure analytical rigour.^50^ The first phase will involve familiarisation with the data by repeatedly listening to the digital recordings while (re)reading the transcripts. The second and third phases will involve identifying patterns and meanings, organising these into initial codes, and generating broad themes and subthemes. The fourth phase will involve reviewing the dataset to ensure themes are coherent and supported by the data. Then in the fifth phase, the themes and subthemes will be refined and developed. During the sixth phase, the themes will be integrated, as it applies to each site. Additionally, descriptive statistics will be calculated using the quantitative data collected via the questionnaires, and subgroup analyses will be conducted, as required.

### Working with priority populations

#### Aboriginal and/or Torres Strait Islander families

In the Taree area, the Biripi Aboriginal Corporation Medical Centre has provided healthcare in a culturally safe, Aboriginal and/or Torres Strait Islander controlled Hub for decades. Following consultation with the Biripi Aboriginal Corporation Medical Centre (ACMC), other local Aboriginal controlled organisations and Biripi Elders, the Ngarra Wakulda ACCG was formed to oversee the codesign process in Taree, with two investigators added to the project from Biripi ACMC. Furthermore, the study has academic oversight provided by Indigenous academics. The process of ensuring the study meets the AH&MRC criteria has resulted in a year of consultation and partnership with the Ngarra Wakulda ACCG. This ensures the codesign process in Taree addresses key issues of: truth-telling and healing; cultural centricity; self-determination; equity; and cultural safety. The AH&MRC identifies some of these central building blocks in the NSW Health Plan 2024–2034.^51^ This cultural safety provides the opportunity for the powerful voice of the local community to be heard, paving the way for systemic change.

A core aim in this codesign process is to develop long standing relationships between the community and the Hub, that allows for meaningful and ongoing community involvement. We are also committed to addressing literacy barriers in research participation. For example, we will adopt approaches such as verbally explaining participant information, besides written participant information sheets, reducing reliance on written materials.

#### Multicultural families

When working with families from CALD and refugee backgrounds to codesign a culturally safe care pathway, it is essential to address the diverse cultural values, beliefs, and practices that influence their healthcare experiences.^52^ Multicultural families bring a rich array of perspectives that must be integrated into the care pathway to ensure it is relevant, respectful, and effective. It is important to consider potential barriers, such as language differences, general and health literacy, and varying levels of access to healthcare services^53^ early when planning a codesign protocol. This enables the participation of families from CALD backgrounds. Hence, providing translated PISCFs that are easy to read, visually engaging and available in various formats (digital, video, hard copy or poster) along with using interpreter services, can aid inclusivity and accessibility. Additionally, the quality of relationships with the researchers can shape experience and engagement,^54^ ensuring that care pathways are accessible, inclusive, and tailored to meet their needs.^55^

### Families living in low socioeconomic areas

When working with families from low socioeconomic backgrounds to codesign an accessible care pathway, it is essential to consider the broader social determinants of health that influence these families’ daily lives and are grounded in the realities of their’ circumstances.^56^ This collaborative approach will address healthcare inequities by integrating the voices of families from low socioeconomic backgrounds and tailoring the tiered care pathway to meet the specific needs of these families, ensuring they receive care that is effective and appropriate.

### Consumer and community involvement

**Figure 5:**
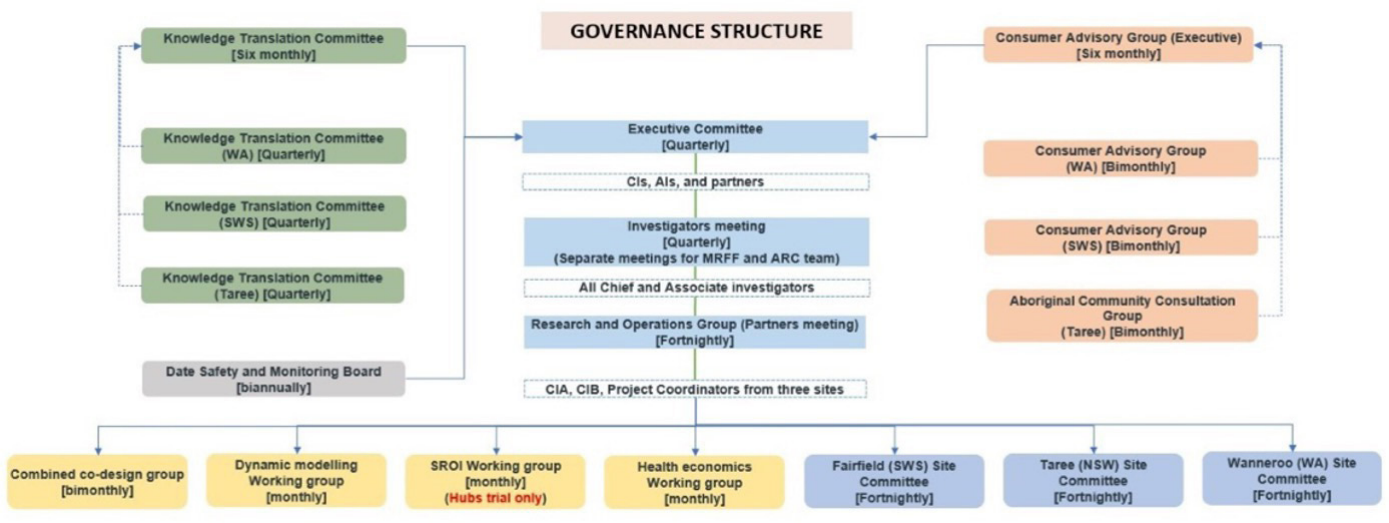
STARS for Kids programme governance structure. AIs, associate investigators; ARC, Australian Research Council; CIA, Chief Investigator A; CIB, Cheif Investigator B; CIs, chief investigators; MRFF, Medical Research Future Fund; NSW, New South Wales; SROI, social return on investment; STARS, Strength-based, Tiered, Accessible Resources and Supports; SWS, South Western Sydney; WA, Western Australia.

Consumer and community involvement is integral to the STARS for Kids programme which is reflected in the governance structure comprising several subcommittees (see figure 5). The steering committee, consisting of the chief invesitgators (CIs) and associate investigators (AIs), will meet quarterly along with project staff to oversee the operation of the study. Furthermore, a research operation group, consisting of project coordinators at each site, will meet fortnightly to update the research activities. A management committee with representatives from the partner organisations along with additional experts co-opted to the project and stakeholders, including consumer representatives (eg, parents/carers, service providers, community leaders - Ngarra Wakulda ACCG) will meet quarterly to consider all the available evidence to develop and refine study protocols and make recommendations.

## Discussion

The STARS for Kids programme will address fragmented and siloed service delivery by integrating healthcare with early childhood education and social care using a tiered care model including digital and place-based Hubs. Codesigning the STARS for Kids programme with families, service providers and community leaders will help to refine the model so that early identification strategies and interventions for child development could be optimised. We anticipate that the close collaboration with families and key stakeholders will address a critical translation gap and result in better engagement of families from priority groups and disadvantaged communities in research and help shape service planning. It will also enable them to contribute to the design and ongoing feedback of the tiered care model to ultimately improve child and family well-being. It is expected that the findings will be critical in providing important insights into the barriers and facilitators of access and engagement with services for priority families. This will also help create a blueprint and refine strategies for successful implementation and scale-up of a strength-based programme for identifying and addressing the child and family needs using a tiered care model including a hybrid digital and/or place-based Hubs in the first 2000 days.

## References

[1] Eapen V, Woolfenden S, Prescott S (2020). Starting at the beginning.

[2] Mendoza Diaz A, Brooker R, Cibralic S (2023). Adapting the ‘First 2000 Days maternal and child healthcare framework’ in the aftermath of the COVID-19 pandemic: ensuring equity in the new world. Aust Health Rev.

[3] Ani C, Ola B, Hodes M (2024). Editorial: Equity, diversity and inclusion in child and adolescent mental health – equality of opportunities should be every child’s right and every society’s obligation. *Child Adoles Ment Health*.

[4] Balmer NJ, Pleasence P, McDonald H (2024). A new perspective on legal need and legal capability.

[5] Woolfenden S, Galea C, Badland H (2020). Use of health services by preschool-aged children who are developmentally vulnerable and socioeconomically disadvantaged: testing the inverse care law. J Epidemiol Community Health.

[6] Woolfenden S, Galea C, Badland H (2019). The intersection of developmental vulnerability and socioeconomic disadvantage on access to health care for preschool aged children: evidence for the inverse-care law. J Epidemiol Community Health.

[7] Campbell F, Conti G, Heckman JJ (2014). Early childhood investments substantially boost adult health. Science.

[8] Carneiro P, Cattan S, Conti G (2025). The short-and medium-term effects of Sure Start on children’s outcomes. The Institute for Fiscal Studies.

[9] Karoly LA, Kilburn MR, Cannon JS (2005). Proven benefits of early childhood interventions.

[10] Eapen V, Woolfenden S, Williams K (2014). “Are you available for the next 18 months?” - methods and aims of a longitudinal birth cohort study investigating a universal developmental surveillance program: the ‘Watch Me Grow’ study. BMC Pediatr.

[11] Woolfenden S, Eapen V, Jalaludin B (2016). Prevalence and factors associated with parental concerns about development detected by the Parents’ Evaluation of Developmental Status (PEDS) at 6-month, 12-month and 18-month well-child checks in a birth cohort. BMJ Open.

[12] Schmied V, Fowler C, Rossiter C (2014). Nature and frequency of services provided by child and family health nurses in Australia: results of a national survey. Aust Health Rev.

[13] Edwards K, Rimes T, Smith R (2020). Improving Access to Early Childhood Developmental Surveillance for Children from Culturally and Linguistically Diverse (CALD) Background. Int J Integr Care.

[14] Kamis C (2021). The Long-Term Impact of Parental Mental Health on Children’s Distress Trajectories in Adulthood. Soc Ment Health.

[15] Smith TA, Kievit RA, Astle DE (2023). Maternal mental health mediates links between socioeconomic status and child development. *Curr Psychol*.

[16] National Mental Health Commission (2021). National children’s mental health and wellbeing strategy 2021.

[17] Productivity Commission (2020). PC productivity insights: Australia’s long term productivity experience.

[18] Mei C, Nelson B, Hartmann J (2020). Transdiagnostic early intervention, prevention, and prediction in psychiatry. Personalized Psychiatry.

[19] Biripi Aboriginal Corporation Medical Centre (2020). Nyiirunba Yabang (our path) 2020-2023. Biripi ACMC.

[20] Australian Commission on Safety and Quality in Health Care (2017). National safety and quality health service standards: user guide for aboriginal and torres strait islander health.

[21] Cibralic S, Alam M, Mendoza Diaz A (2022). Utility of screening for adverse childhood experiences (ACE) in children and young people attending clinical and healthcare settings: a systematic review. BMJ Open.

[22] Kelly-Irving M, Delpierre C (2019). A Critique of the Adverse Childhood Experiences Framework in Epidemiology and Public Health: Uses and Misuses. Soc Policy Soc.

[23] Masten AS (2001). Ordinary magic. Resilience processes in development. Am Psychol.

[24] Sege RD (2021). Reasons for HOPE. Pediatrics.

[25] Baker J, Kohlhoff J, Onobrakpor S-I (2020). The Acceptability and Effectiveness of Web-Based Developmental Surveillance Programs: Rapid Review. JMIR Mhealth Uhealth.

[26] Kohlhoff J, Dadich A, Varghese J (2022). Consumer and health professional perceptions of Watch Me Grow - Electronic (WMG-E) platform for developmental surveillance in early childhood: A qualitative study. Aust J Gen Pract.

[27] Hoang N-P, Ma T, Silverwood AJ (2024). Place-based approach to support children’s development towards sustainable development goals: A scoping review of current effort and future agenda. Child Youth Serv Rev.

[28] Glover J, Samir N, Kaplun C (2021). The effectiveness of place-based interventions in improving development, health and wellbeing outcomes in children aged 0–6 years living in disadvantaged neighbourhoods in high-income countries – A systematic review. *Wellbeing, Space and Society*.

[29] lnstitute for Fiscal Studies (2024). The short- and medium-term impacts of Sure Start on educational outcomes.

[30] Bazzano AN, Martin J, Hicks E (2017). Human-centred design in global health: A scoping review of applications and contexts. PLoS One.

[31] McIlduff C, Forster M, Carter E (2020). Model of engaging communities collaboratively: working towards an integration of implementation science, cultural adaptation and engagement. Int J Crit Indig Stud.

[32] Nelson HJ, Angus B, Munns A (2023). Models, theoretical design and formal evaluation of integrated specialist community health service provision for the first 2000 days: a scoping review. BMJ Open.

[33] Rodrigues De Sousa Junior R, Oliveira Souto D, Ribeiro Ferreira F (2024). Parents’ perceptions of a modified sports intervention for children with cerebral palsy. Develop Med Child Neuro.

[34] Noble K, Hurley P, Macklin S (2020). COVID-19, employment stress and student vulnerability in Australia.

[35] Commonwealth of Australia (2024). Australian early development census national report 2024.

[36] Winata T, Smead M, Cibralic S Watch Me Grow-electronic (WMG-E) surveillance approach to identify and address child development, parental mental health and psychosocial needs. Aust N Z J Psychiatry.

[37] Eapen V, Woolfenden S, Schmied V (2021). “Watch Me Grow- Electronic (WMG-E)” surveillance approach to identify and address child development, parental mental health, and psychosocial needs: study protocol. BMC Health Serv Res.

[38] Barr KR, Hawker P, Winata T (2024). Family member and service provider experiences and perspectives of a digital surveillance and service navigation approach in multicultural context: a qualitative study in identifying the barriers and enablers to Watch Me Grow-Electronic (WMG-E) program with a culturally diverse community. BMC Health Serv Res.

[39] Barbaro J, Winata T, Gilbert M (2023). General practitioners’ perspectives regarding early developmental surveillance for autism within the australian primary healthcare setting: a qualitative study. *BMC Prim Care*.

[40] Karlov L, Masi A, Diaz AM (2025). A Preliminary Trial of an Early Surveillance Program for Autism and Developmental Delays within General Practices. J Dev Phys Disabil.

[41] Abookire S, Plover C, Frasso R (2020). Health Design Thinking: An Innovative Approach in Public Health to Defining Problems and Finding Solutions. Front Public Health.

[42] Bessarab D, Ng’andu B (2010). Yarning About Yarning as a Legitimate Method in Indigenous Research. *IJCIS*.

[43] Damschroder LJ, Reardon CM, Widerquist MAO (2022). The updated Consolidated Framework for Implementation Research based on user feedback. Implement Sci.

[44] Hodgins M, Ostojic K, Hu N (2022). Study protocol for a real-world evaluation of an integrated child and family health hub for migrant and refugee women. BMJ Open.

[45] Taylor MJ, McNicholas C, Nicolay C (2014). Systematic review of the application of the plan–do–study–act method to improve quality in healthcare. *BMJ Qual Saf*.

[46] Weiner BJ, Lewis CC, Stanick C (2017). Psychometric assessment of three newly developed implementation outcome measures. Implement Sci.

[47] Castleden H, Morgan VS, Franks A (2016). Practicing qualitative methods in health geographies.

[48] Samaranayake P, Dadich A, Fitzgerald A (2016). Developing an evaluation framework for clinical redesign programs: lessons learnt. J Health Organ Manag.

[49] Guest G, Bunce A, Johnson L (2006). How many interviews are enough? An experiment with data saturation and variability. Field methods.

[50] Braun V, Clarke V (2006). Using thematic analysis in psychology. Qual Res Psychol.

[51] NSW Ministry of Health (2024). NSW Health Plan 2024-2034: sharing power in system reform.

[52] Betancourt JR, Green AR, Carrillo JE (2003). Defining cultural competence: a practical framework for addressing racial/ethnic disparities in health and health care. Public Health Rep.

[53] Bonakdar Tehrani M, Blythe S, Trajkovski S (2024). Co-Design Model of Support for Child and Family Health Nurse Practice with Culturally and Linguistically Diverse Families. IJERPH.

[54] O’Brien J, Fossey E, Palmer VJ (2021). A scoping review of the use of co‐design methods with culturally and linguistically diverse communities to improve or adapt mental health services. *Health Soc Care Community*.

[55] Andermann A, CLEAR Collaboration (2016). Taking action on the social determinants of health in clinical practice: a framework for health professionals. CMAJ.

[56] Braveman P, Egerter S, Williams DR (2011). The social determinants of health: coming of age. Annu Rev Public Health.

