# Supplementary material for: Codesign and knowledge translation of the Strength-based, Tiered, Accessible Resources and Supports (STARS) for Kids study to identify and support child development, parental mentalwell-being and family psychosocial needs: a mixed-methods research protocol
Source: BMJ Paediatr Open. 2026 May 7;10(1):e004031. doi: 10.1136/bmjpo-2025-004031 (PMC13157746; doi:10.1136/bmjpo-2025-004031)
Supplement: Supplementary file 1 [file bmjpo-2025-004031.reviewer_comments.pdf]

PEER REVIEW HISTORY

BMJ Paediatrics Open publishes all reviews undertaken for accepted manuscripts. Reviewers are asked to complete a checklist review form and are provided with free text boxes to elaborate on their assessment. These free text comments are reproduced below.

ARTICLE DETAILS

**TITLE (PROVISIONAL)**	Codesign and knowledge translation of the Strength-based, Tiered, Accessible Resources and Supports (STARS) for Kids study to identify and support child development, parental mental wellbeing, and family psychosocial needs - A mixed-methods research protocol
**AUTHORS**	John, James R.<br>Robinson, Hayley<br>Lambiase, Sini<br>Nicholls, Sophie<br>Diez, Maite<br>Barr, Karlen R<br>Ostojic, Katarina<br>Doyle, Aunty Kerrie<br>Kliendienst, Luana<br>Foster, Melissa<br>Syron, Lynette<br>Carson, Toni<br>Kohlhoff, Jane<br>Dadich, Ann<br>Brennan, Clare<br>Katsamangos, Bree<br>Kemp, Lynn<br>Finlay-Jones, Amy<br>O'loughlin, Grainne<br>Boydell, Katherine<br>Raman, Shanti<br>Deen, Nicole<br>Lawson, Kenny<br>Schmied, Virginia<br>Katz, Ilan<br>Page, Andrew<br>Grace, Rebekah<br>Kanaan, Anna-Marie<br>Young, Rebecca<br>Brandtman, Erin<br>Bratel, Lee<br>hodgins, michael<br>Lingam, Raghu<br>Lam-Cassettari, Christa<br>Goldfeld, Sharon<br>Walker, Adam K<br>Lin, Ping I<br>Morton, Susan<br>Silva, Desiree<br>Downs, Jenny<br>Woolfenden, Susan<br>Eapen, Valsamma<br>Aboriginal Community Consultation Group, on behalf of<br>STARS for Kids research group, on behalf of

VERSION 1 – REVIEW

**REVIEWER NAME**	**Sinopoulou, Vasiliki**
**REVIEWER AFFILIATION**	University of Lancashire, School of Medicine
**REVIEWER CONFLICT OF INTEREST**	No Conflict of Interest
**AI DISCLOSURE**	No, I have not used AI
**DATE REVIEW RETURNED**	19-Jan-2026

**GENERAL COMMENTS**	This is a very well-written and well-designed protocol. A couple of minor comments:<br><br>1. "Challenges in the patient journey": Will the programme be targeted towards children facing specific health challenges, having or seeking diagnoses, i.e. "patients"? If not, maybe you can use a different word instead of "patients".<br>2. "Strength-based": I think from the context I understand what you mean by "strength-based programme", but because it's in your title and acronym you could have a paragraph or sentence where you explain it explicitly.<br>3. "Yarning": I am not familiar with this concept. It could be useful to briefly explain.<br>4. "Evidence-based practices": How will these be identified?<br>5. The "bring an artefact" and online artwork exhibition are excellent ideas. I am a little unclear as to how they fit in the model evaluation phase and if the artefacts will represent past service experiences, ideal service experiences, or the participants experience with co-designing?<br><br>Figure 1 is mostly about Phase 2 and the trials that are planned to follow, the design of which presumably depends on the Phase 1 co-design. I think it would be more appropriate if the figure presented schematically Phase 1 instead of Phase 2, and only briefly mentioned Phase 2 as future plans.<br><br>Figure 2 would benefit from a heading above the green section and another above the yellow section.

**REVIEWER NAME**	**Spencer, Nick**
**REVIEWER AFFILIATION**	University of Warwick Warwick Medical School, Division of Mental Health and Wellbeing
**REVIEWER CONFLICT OF INTEREST**	No Conflict of Interest
**AI DISCLOSURE**	No, I have not used AI
**DATE REVIEW RETURNED**	24-Jan-2026

**GENERAL COMMENTS**	The inverse care law states that those most in need of health care and other services are least likely to receive them. This law lies at the root of the study proposed in this paper. As the authors state " Many children and their families, especially those from priority populations, experience barriers to accessing high quality early childhood health, education, social, and legalservices. " The ambitious and laudable aim of the project is to "co-design with families a program to optimise child development, parental mental wellbeing, and family psychosocial needs in the first 2000 days from

pregnancy to start of school." This is a major undertaking and, while I fully support the aim of reducing barriers to essential services, I have some concerns re aspects of the protocol.

1. The protocol states that "the goal is to identify early and facilitate tailored supports for child development, parental mental health, and family psychosocial needs" (p.10 lines 47-49). The authors identify the siloed nature of service delivery and the fragmentation, duplication and service gaps in the current Australian child and family service system and there is little doubt that a service designed by parents specifically to meet their needs, as is envisaged by this study, would make it easier for parents to use the services and benefit their children and themselves. Unfortunately, early identification of child developmental problems is not straightforward and caution is needed in predicting likely success of this goal (see Oberklaid et al 2013 - https://doi.org/10.1136/archdischild-2013-304091). The study proposes to use the Watch Me Grow-Electronic (WMG-E) platform, a digital data collection tool that will be used by parents to make a series of assessments of their children's developmental needs (as well as parental needs). These will be reviewed at 'relevant developmental checks'. I was able to download reference 15, a qualitative study of consumer and health professionals perceptions of WMG but couldn't find reference 25. If WMG-E is the instrument by which child developmental problems are identified, it is essential that the authors provide evidence of its effectiveness and validity.

2. A related concern to that discussed in point 1 above is uptake of well child and development checks. As has been noted in many countries, uptake of these checks in Australia is low. Professor Eapen's submission to a 2024 NSW Government Inquiry states "Despite substantial investment in state and national early childhood education and care programs, uptake remains low, with only 20-30% of families accessing routine health and developmental services in certain regions. Children from disadvantaged backgrounds often miss recommended developmental checks, highlighting significant inequity" (https://www.parliament.nsw.gov.au/ladocs/submissions/83639/Submission%2024%20-%20Professor%20Valsamma%20Eapen.pdf). Given the areas chosen for the proposed research, it is safe to predict that uptake of the 'relevant developmental checks' will be similarly low with the result that many children with developmental problems will be missed. Part of the aim of the study is to overcome barriers but the protocol should specifically address this issue of uptake and how the project will address it.

3. The methodology of the qualitative elements of the study is extensively detailed and is generally robust. However, eligible participants are self-selected from adverts in a range of services including those that are known to have poor uptake (see point 2). A potential concern is that the most vulnerable and 'hard to reach' (see experience from Sure Start in the UK – Coe et al doi:10.1111/j.1365-2214.2008.00816.x) will be under-represented among study participants. The protocol makes some passing reference to barriers to service access among different study target groups but the authors should consider including a section on how selection of 'hard to reach' participants will be addressed.

In summary, this study protocol is addressing an important problem in the delivery and effectiveness of services for vulnerable children in the first 2000 days of their lives in Australia. If the results of this study can contribute to a better understanding among health, education and other service providers of the needs of families and their children in relation to developmental issues, then it will make a

	valuable contribution to research in this area. However, I think the protocol will be strengthened if the authors specifically address some of the issues outlined above.<br>Nick Spencer

VERSION 1 – AUTHOR RESPONSE

Reviewer: 1

Ms. Vasiliki Sinopoulou, University of Lancashire

Comments to the Author

This is a very well-written and well-designed protocol. A couple of minor comments:

1. "Challenges in the patient journey": Will the programme be targeted towards children facing specific health challenges, having or seeking diagnoses, i.e. "patients"? If not, maybe you can use a different word instead of "patients".

Author response: We thank the reviewer for the feedback. The STARS for Kids program is not limited to children with diagnosed conditions or those actively seeking healthcare, but rather aims to support children and families where they are in the community, including prevention and early support. We have therefore replaced the term “patient journey” to “child and family service journey” to better reflect a family- and community-centred perspective (Page 10)

Modifications: The term “…challenges in the patient journey” has been changed to “…challenges in the child and family service journey”.

2. "Strength-based": I think from the context I understand what you mean by "strength-based programme", but because it's in your title and acronym you could have a paragraph or sentence where you explain it explicitly.

Author response: We agree and have added a concise definition of “strength-based program” to articulate how this concept underpins the STARS for Kids program (Page 9).

Modifications: “Additionally, research on the first 2000 days of life has largely focused on risks such as adverse childhood experiences (ACEs), but emerging evidence highlights their limitations for identifying individual vulnerabilities, prompting a shift towards strengths-based approaches to better support families and improve long-term outcomes. Strengths-based programs recognise and build on the existing capacities, resilience, cultural knowledge, and resources of children, families, and communities, thereby emphasising empowerment, partnership, and respect for lived experience in the design and delivery of supports”.

3. "Yarning": I am not familiar with this concept. It could be useful to briefly explain.

Author response: We have now added a brief explanation of yarning to ensure clarity for readers unfamiliar with Indigenous research methodologies (Page 15).

Modifications: “Yarning is an Indigenist research method that involves informal, narrative-based conversations with Aboriginal and Torres Strait Islander families that is culturally appropriate and grounded in relationality, trust-building, and active listening”.

4. "Evidence-based practices": How will these be identified?

Author response: Evidence-based practices will be identified and integrated during the ideation phase, combining published evidence with local knowledge and stakeholder expertise. We have expanded this in the ‘Phase 3: Creating new ideas’ section (Page 15).

Modifications: “This phase will draw on evidence-based practices identified through mapping existing local services and consultation with clinical, community, and policy experts, integrating these with new insights to develop a tiered model of care.”

5. The "bring an artefact" and online artwork exhibition are excellent ideas. I am a little unclear as to how they fit in the model evaluation phase and if the artefacts will represent past service experiences, ideal service experiences, or the participants experience with co-designing?

Author response: The artefacts may represent any of the following: past service experiences, participants’ ideal vision of integrated care, or their experiences of co-design. They will be used primarily as a qualitative sense-making and knowledge translation tool within the model evaluation phase (Page 18).

Modifications: “To visualise service experiences, the interviewees will be invited to bring an artefact to the interview that may capture their: past experiences; ideal vision that epitomises integrated care via digital/place-based Hubs to them (e.g., material objects, photographs, equipment, artwork); or their experience with codesign, reflecting a modified form of photovoice; discuss why they chose the artefact and what it represents to them; and map client journeys to ascertain patterns and clarify how the service system responds (or can respond to) family needs and preferences”.

6. Figure 1 is mostly about Phase 2 and the trials that are planned to follow, the design of which presumably depends on the Phase 1 co-design. I think it would be more appropriate if the figure presented schematically Phase 1 instead of Phase 2, and only briefly mentioned Phase 2 as future plans.

Author response: We agree and have revised Figure 1 and its legend so that it primarily presents Phase 1 (codesign), with Phase 2 clearly identified as a future implementation and evaluation phase.

Modifications: Figure 1 has been modified as suggested.

7. Figure 2 would benefit from a heading above the green section and another above the yellow section.

Author response: We have revised Figure 2 to include headings to improve readability.

Modifications: Figure 2 has been modified as suggested.

Reviewer: 2
Dr. Nick Spencer, University of Warwick Warwick Medical School

Comments to the Author
The inverse care law states that those most in need of health care and other services are least likely to receive them. This law lies at the root of the study proposed in this paper. As the authors state “Many children and their families, especially those from priority populations, experience barriers to

accessing high quality early childhood health, education, social, and legal services.” The ambitious and laudable aim of the project is to “co-design with families a program to optimise child development, parental mental wellbeing, and family psychosocial needs in the first 2000 days from pregnancy to start of school.” This is a major undertaking and, while I fully support the aim of reducing barriers to essential services, I have some concerns re aspects of the protocol.

1. The protocol states that “the goal is to identify early and facilitate tailored supports for child development, parental mental health, and family psychosocial needs” (p.10 lines 47-49). The authors identify the siloed nature of service delivery and the fragmentation, duplication and service gaps in the current Australian child and family service system and there is little doubt that a service designed by parents specifically to meet their needs, as is envisaged by this study, would make it easier for parents to use the services and benefit their children and themselves. Unfortunately, early identification of child developmental problems is not straightforward and caution is needed in predicting likely success of this goal (see Oberklaid et al 2013 - https://doi.org/10.1136/archdischild-2013-304091). The study proposes to use the Watch Me Grow-Electronic (WMG-E) platform, a digital data collection tool that will be used by parents to make a series of assessments of their children’s developmental needs (as well as parental needs). These will be reviewed at ‘relevant developmental checks’. I was able to download reference 15, a qualitative study of consumer and health professionals perceptions of WMG but couldn’t find reference 25. If WMG-E is the instrument by which child developmental problems are identified, it is essential that the authors provide evidence of its effectiveness and validity.

Author response: We thank the reviewer for this feedback. We have strengthened the description of the Watch Me Grow-Electronic (WMG-E) platform by highlighting its demonstrated feasibility for engaging and empowering priority populations, acceptability to service providers, and effectiveness in identifying developmental conditions, including autism, with high sensitivity, citing relevant prior work (Page 13).

Modifications: “The WMG-E has demonstrated feasibility for engaging and empowering priority populations,19 30 acceptability for service providers,31 and effectiveness in identifying developmental conditions including autism32 with high sensitivity (94% against gold-standard assessment).”

2. A related concern to that discussed in point 1 above is uptake of well child and development checks. As has been noted in many countries, uptake of these checks in Australia is low. Professor Eapen’s submission to a 2024 NSW Government Inquiry states “Despite substantial investment in state and national early childhood education and care programs, uptake remains low, with only 20-30% of families accessing routine health and developmental services in certain regions. Children from disadvantaged backgrounds often miss recommended developmental checks, highlighting significant inequity” (https://www.parliament.nsw.gov.au/ladocs/submissions/83639/Submission%2024%20-%20Professor%20Valsamma%20Eapen.pdf). Given the areas chosen for the proposed research, it is safe to predict that uptake of the ‘relevant developmental checks’ will be similarly low with the result that many children with developmental problems will be missed. Part of the aim of the study is to overcome barriers but the protocol should specifically address this issue of uptake and how the project will address it.

Author response: We thank the reviewer for highlighting this important point regarding the low and inequitable uptake of child developmental checks. We have addressed this feedback in both the Introduction and Methods sections. In the Introduction, we now explicitly note that systematic mechanisms to engage children and families early are lacking, as reflected in the low completion of developmental checks beyond six months of age. In the Methods, we clarify that the WMG-E platform is designed to reach families in their homes or communities by leveraging opportunistic service contacts such as childhood immunisations with high coverage and through trusted providers,

including primary care, multicultural, refugee, and Aboriginal services, as well as community hubs such as libraries and playgroups, thereby directly addressing barriers to uptake (Pages 7 and 12).

Modifications:
Introduction section – "Although the benefits of early interventions are well evidenced, systematic mechanisms to engage children and families early are still lacking, as reflected in the low and inequitable completion of developmental checks beyond six months of age."

STARS for Kids conceptual model – "The WMG-E will reach families in their homes or in the community on the principle of 'going to where the children and families go' and leveraging opportunistic service contacts (e.g., childhood immunisation that has 95% reach/uptake), facilitated through trusted providers (e.g., primary care, multicultural, refugee or Aboriginal services, and other community services such as libraries and playgroups)."

3. The methodology of the qualitative elements of the study is extensively detailed and is generally robust. However, eligible participants are self-selected from adverts in a range of services including those that are known to have poor uptake (see point 2). A potential concern is that the most vulnerable and 'hard to reach' (see experience from Sure Start in the UK – Coe et al doi:10.1111/j.1365-2214.2008.00816.x) will be under-represented among study participants. The protocol makes some passing reference to barriers to service access among different study target groups, but the authors should consider including a section on how selection of 'hard to reach' participants will be addressed.

Author response: We thank the reviewer for this observation. We have added a dedicated subsection outlining strategies to engage families who are traditionally under-represented in research and services (Page 12).

Modifications: "To minimise under-representation of families who are traditionally described as 'hard to reach', recruitment will be supported by attendance at community outreach programs or in-person engagement within trusted community hub providers and organisations, Aboriginal Community Controlled Organisations, and frontline service providers. Additionally, flexible participation options (in-person, telephone, and online), alongside the use of interpreters where required, will be employed to support inclusive participation."

In summary, this study protocol is addressing an important problem in the delivery and effectiveness of services for vulnerable children in the first 2000 days of their lives in Australia. If the results of this study can contribute to a better understanding among health, education and other service providers of the needs of families and their children in relation to developmental issues, then it will make a valuable contribution to research in this area. However, I think the protocol will be strengthened if the authors specifically address some of the issues outlined above.

Author response: We thank the reviewer for their supportive summary. We believe the revisions directly address the issues raised and strengthen the protocol's transparency, feasibility, and equity focus.
